# Heat Transfer Enhancement Using Al_2_O_3_-MWCNT Hybrid-Nanofluid inside a Tube/Shell Heat Exchanger with Different Tube Shapes

**DOI:** 10.3390/mi14051072

**Published:** 2023-05-18

**Authors:** Maissa Bouselsal, Fateh Mebarek-Oudina, Nirmalendu Biswas, Abdel Aziz I. Ismail

**Affiliations:** 1 Department of Physics, Faculty of Sciences, University of 20 Août 1955-Skikda, Skikda 21000, Algeria; 2 Laboratoire des Matériaux et Génie Energétique (LMGE), University of 20 Août 1955-Skikda, Skikda 21000, Algeria; 3 Department of Power Engineering, Jadavpur University, Salt Lake, Kolkata 700106, India; 4 Mechanical Engineering Department, College of Engineering and Islamic Architecture, Umm Al-Qura University, Makkah P.O. Box 5555, Saudi Arabia

**Keywords:** heat exchanger, hybrid nanofluid, tube shapes, entropy generation, heat transfer

## Abstract

The high demand for compact heat exchangers has led researchers to develop high-quality and energy-efficient heat exchangers at a lower cost than conventional ones. To address this requirement, the present study focuses on improvements to the tube/shell heat exchanger to maximize the efficiency either by altering the tube’s geometrical shape and/or by adding nanoparticles in its heat transfer fluid. Water-based Al_2_O_3_-MWCNT hybrid nanofluid is utilized here as a heat transfer fluid. The fluid flows at a high temperature and constant velocity, and the tubes are maintained at a low temperature with various shapes of the tube. The involved transport equations are solved numerically by the finite-element-based computing tool. The results are presented using the streamlines, isotherms, entropy generation contours, and Nusselt number profiles for various nanoparticles volume fraction 0.01 ≤ *φ* ≤ 0.04 and Reynolds numbers 2400 ≤ Re ≤ 2700 for the different shaped tubes of the heat exchanger. The results indicate that the heat exchange rate is a growing function of the increasing nanoparticle concentration and velocity of the heat transfer fluid. The diamond-shaped tubes show a better geometric shape for obtaining the superior heat transfer of the heat exchanger. Heat transfer is further enhanced by using the hybrid nanofluid, and the enhancement goes up to 103.07% with a particle concentration of 2%. The corresponding entropy generation is also minimal with the diamond-shaped tubes. The outcome of the study is very significant in the industrial field and can solve many heat transfer problems.

## 1. Introduction

Solar energy has become the energy of the times and the only solution for the poor to replace petroleum products. Heat exchangers are used in diverse fields of application such as in water heating systems in solar collectors, solar thermal energy storage systems, and other compact heat exchanger applications. The efficiency of heat exchangers is related to metallic and non-metallic materials such as aluminum, iron, copper, steel, bronze, ceramic and glass PTFE, and graphite. The heat transfer coefficient varies from one material to another. Therefore, by increasing the heat transfer coefficient, the heat exchange capacity increases. This is accomplished by modifying the geometric shape of the heat exchangers. The importance of heat exchangers differs from field to field with increasing demand; the purpose of using them is to save energy and reduce the cost. Thus, the heat exchanger is an essential element of any energy management policy and therefore of environmental protection. Indeed, a large part of the energy used in the various sectors passes at least once through a heat exchanger, which makes it a key device for thermal engineers and essential in energy management [1,2]. In general, heat exchange between the two fluids is accomplished by the heat exchangers [3] and the same versatile application in various industries for heating or cooling [4].

With the development of science and technology, energy guidance has become a major goal for scientists due to their urgent need to provide natural energy sources. Where many companies and factories compete to rationalize energy consumption through regenerative heat exchangers, it is a weapon of the times that saves large amounts of wasted energy. Thanks to it, it can capture as much waste heat as possible and use it to heat the cold gases that are usually heated in thermal furnaces and engines that use fossil fuels to warm up. Thus, these motors use less energy and therefore consume valuable fossil fuel. Underground heat exchangers have also been used underground to take advantage of geothermal heat from the earth in cooling and heating fluids for industrial, residential, and agricultural uses.

These devices are specially designed to activate the vital act of various thermal and chemical processes through the transfer of heat from one liquid to another at different temperatures. The design of these heat exchangers with reliable operation of the systems is mainly adopted for the transfer of heat in spite of the problems of this reactor such as cooling of the components of the system to reuse and avoid burning it and to intensify these exchanges to improve the production rate. To improve the heat transfer using convection as a cooling method, the researchers conducted numerical and experimental tests about the nature of the systems in which they occur and the properties of the fluids involved (physico-chemical properties).

There are different types of heat exchangers, and thus, the selection of the right type for a particular process is very important because incorrect selection leads to the malfunctioning of the system operation or sometimes failure of the equipment. Amongst the different types of heat exchangers [3,5], tube and shell types are most used in several process industries [6]. This is due to the ease of changing and cleaning these parts; it also enables high-pressure applications. It is used in many fields such as oil refineries, chemical processing, the electrical industry, hydrocarbon processing units, feed water heaters, condensing units, and others. Therefore, there is a continual demand for the development of compact heat exchangers depending on the application. To meet these demands, researchers are continually paying attention to further development in heat exchange efficacy. Apart from the geometric modification of the heat transfer surfaces/unit, there is a promising technique for further improvement in the efficacy of heat transfer by adding nano-sized particles (single type or multi types) in the heat transfer fluids. The mixture of such nanoparticles and heat transfer fluid is termed nanofluid/hybrid nanofluids. Studies have shown that adding nanoparticles to a carrier liquid matrix can increase the heat transfer compared to a pure assembled fluid, or that hybrid nanofluids have particular physical and chemical properties such as high thermal conductivity [7,8,9,10,11].

Actual experiments and their analysis are usually expensive as well as time-consuming. Therefore, wide variations for the parametric analysis of the prototypes are also not suitable [12,13]. Therefore, computational analysis is preferable to give a clear study of these thermal devices. There is a vast pool of literature on heat exchangers and heat transport improvement analysis [14]. Zaversky et al. [15] analyzed accurate modeling of a heat exchanger. Shahdad and Fazel [16] presented the role of perforated fins instead of simple fins for improving the convective coefficient of heat transfer and the Nusselt number. Xie et al. [17] examined the enhanced heat transfer using honeycomb tubes over smooth tubes. Matos et al. [18,19] compared the heat transfer of elliptical and circular tubes (12 numbers) under various Re values. Nouri-Borujerdi and Lavasani [20,21] examined thermo-fluid flow characteristics of simple cam-shaped conduits. Mohanty et al. [22] performed a 2D analysis in an elliptical circular tube-based heat exchanger. Dharmaiah et al. [23] numerically studied a magnetic dipole effect on a radiative ferromagnetic liquid. Li et al. [24] investigated the heat transfer as well as frictional losses in an elliptical tube-based heat exchanger.

Bouris et al. [25] studied the frictional losses in an elliptical tube-based heat exchanger and found that an increase in the heat transfer area causes a higher pressure drop. Moawed [26] conducted an experimental investigation on the forced convective heat transfer over the surface of a spiral tube-based heat exchanger. Rosen and Dincer [27] found that lower temperature differences enhance the heat exchanger performance. Khan et al. [28] analyzed the forced convective phenomena in elliptical-shaped tubes and found that the transfer of heat is more with a growing air-water flow. Harris and Goldschmidt [29] investigated the consequence of the angle of attack on the overall thermal performance in a heat exchanger with an elliptical-shaped tube. Teo et al. [30] numerically analyzed a finned heat exchanger with elliptical-shaped tubes. They observed 30% enhanced heat transfer in comparison to circular tubes. Li et al. [31] carried out calculations on thermal behavior with elliptical-shaped tubes with an axial ratio of 0.3, 0.5, and 0.8.

Kumar and Jhinge [32] conducted experimental work on tube and shell heat exchangers containing split baffles at different angular orientations of the baffles: 0°, 15°, 30° and 45° from the horizontal. Raj and Ganne [33] investigated the impact of various baffle tilt angles on the heat transfer characteristics of a tube/shell heat exchanger for three different tilt angles of the baffle: 0°, 10° and 20°, with a deflector cut of 36%. Zhang et al. [34] performed a shell/tube heat exchanger simulation with overlapping non-continuous intermediate helical baffles (30°, 40°, and 50° helix angles). Sivarajan et al. [35] performed a numerical simulation of a continuous spiral baffle tube/shell heat exchanger; their results indicated that the spiral baffle tube/shell heat exchanger has better flow performance and better heat transfer than the conventional shell and tube baffle heat exchanger. Kwon et al. [36] showed that with the use of ZnO nanoparticles and Al_2_O_3_, the end results are that the coefficient of heat transfer increases to 30% at a concentration of 6% Al_2_O_3_. Albadr et al. [37] studied heat transfer through a heat exchanger using the nanofluid Al_2_O_3_ at different concentrations. The forced convection heat transfer coefficient is slightly higher than that of the base liquid at the same inlet temperatures and mass flow rate. Very recently, Asadi et al. [38] carried out an experimental study of the heat transfer performance of Al_2_O_3_-MWCNT hybrid nanofluid (oil based) and found an enhanced heat transfer. Ghazanfari et al. [39] numerically analyzed the overall performance of a twisted-tube-based shell-type heat exchanger utilizing Al_2_O_3_ nanoparticles and found a 20% enhanced heat transfer and an increase in the pressure drop up to 40%.

A detailed review of the performance improvement in heat exchangers could be found in refs [40,41,42].

The vast pool of literature clearly indicates the usefulness of heat exchanger analysis due to the diverse fields of application in critical thermal systems. Off-course design of a compact heat exchanger (shell and tube type) is a critical task for the designer for maximizing the heat transfer and lowering the pressure drop. Both of these criteria significantly affect the equipment sizing as well as its cost. Therefore, designing high-quality and energy-efficient heat exchangers at a lower cost than the conventional (circular tube bundle) ones is the key target for the manufacturer. To address this requirement, the present study focuses on the improvements of the tube/shell heat exchanger to maximize the efficiency either by altering the geometrical shape of the tubes and/or by adding nanoparticles in its heat transfer fluid. According to the literature, this work has never been the subject of a previous study. The aim of this work is to find the ideal shape of the tubes for better heat transfer using a hybrid nanofluid and to compare it with a conventional fluid, H_2_O. In this study, four different shapes (circular, square, rectangular, and diamond) of the tube bundles are examined under a wide parametric range of nanoparticles with a volume fraction 0.01 ≤ *φ* ≤ 0.04 and Reynolds numbers of 2400 ≤ Re ≤ 2700. For maximizing the heat transfer, the entropy generation rate is also analyzed systematically. Water-based Al_2_O_3_-MWCNT hybrid nanofluid is utilized here as a heat transfer fluid.

## 2. Geometry Description and Mathematical Model

**Figure 1:**
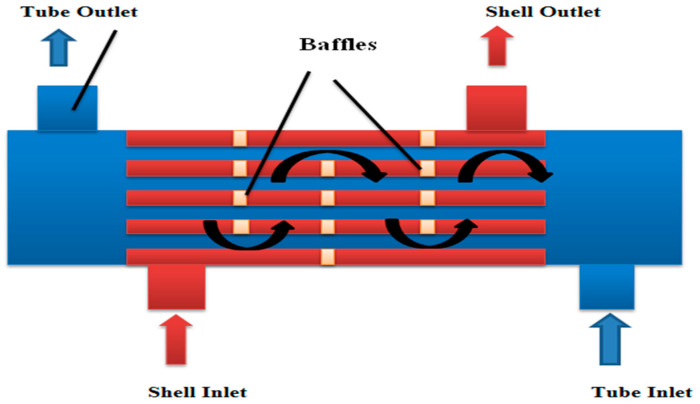
Schematic representation of a conventional tube/shell heat exchanger.

**Figure 2:**
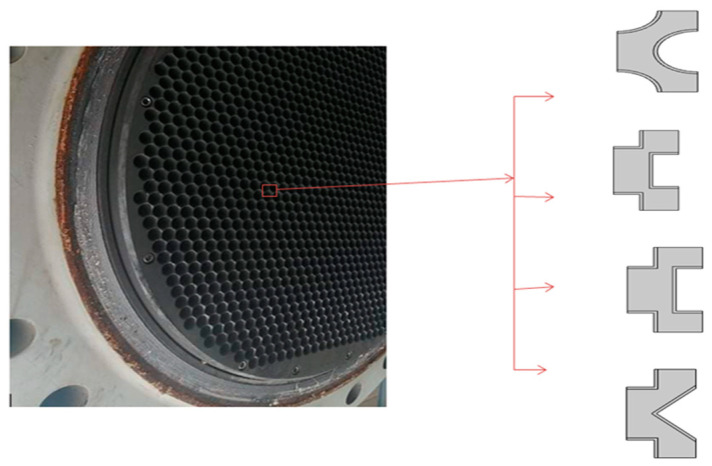
Tube/shell heat exchanger section from RA_2_K SONATRACH Skikda, Algeria.

A schematic representation of a conventional tube/shell heat exchanger (in two-dimensional forms) is shown in Figure 1. Figure 2 shows the actual view of a tube/shell heat exchanger section from RA_2_K SONATRACH Skikda, Algeria. An enlarged view of a tube and its modified shape (circular, square, rectangular, and diamond) are also shown in Figure 2. The circular shape is the basic shape and is taken as a reference for the comparison of betterment in heat transfer 9, if any). The computational domain consists of half of a tube bundle and a quarter portion of the two tubes. The material of the tube bundles is considered to be aluminum. The water-based Al_2_O_3_-MWCNT hybrid-nanofluid flows through the gaps in between the tube bundles and carries the heat from the heated fluid flowing through the tube of different shapes. The purpose of this study is to model the shell and tube heat exchanger to improve the heat transfer of the cooling fluid using the Al_2_O_3_-MWCNT hybrid nanofluid.

The leading equations for the continuity, momentum, and energy are solved numerically. The k-ε turbulent flow model is used to solve this mathematical model [43,44,45], and the expression is given as:

(1) $$\frac{\partial u}{\partial t}+\rho \left(u\cdot \nabla \right)u=\nabla \cdot \left[-pI+K\right]+F$$

(2) $$\frac{\partial \rho }{\partial t}+\nabla \cdot \left(\rho u\right)=0$$

where

(3) $$K=\left(µ+{µ}_{\tau }\right)\left(\nabla u+{\left(\nabla u\right)}^{\tau }\right)-\frac{2}{3}\left(µ+{µ}_{\tau }\right)\left(\nabla \cdot u\right)I-\frac{2}{3}\rho kI$$

(4) $$\rho c_{p}\left(\frac{\partial T}{\partial t}+u\;\cdot \nabla T\right)+\nabla \cdot \left(q+q_{r}\right)=\beta T\left(\frac{\partial p}{\partial t}+u\;\cdot \;\nabla p\right)+\tau :\nabla u+Q$$

$$\beta =-\frac{1}{\rho }\frac{\partial \rho }{\partial T}$$

*Q* contains heat sources other than viscous dissipation (W/m^3^)

$$Q_{p}=\beta T\left(\frac{\partial p}{\partial t}+u\cdot \nabla p\right)$$

The second term represents viscous dissipation in the fluid:

$$Q_{vd}=\tau :\nabla u$$

For the turbulent kinetic energy, we can write the following:

(5) $$\rho \frac{\partial k}{\partial t}+\rho \left(u\cdot \nabla \right)k=\nabla \cdot \left[\left(µ+\frac{{µ}_{\tau }}{\sigma_{k}}\right)\nabla k\right]+p_{k}-\rho ℰ$$

For dissipation:

(6) $$\rho \frac{\partial ℰ}{\partial t}+\rho \left(u\cdot \nabla \right)ℰ=\nabla \cdot \left[\left(µ+\frac{{µ}_{\tau }}{\sigma_{ℰ}}\right)\nabla ℰ\right]+C_{ℰ1}\frac{ℰ}{k}p_{K}-C_{ℰ2}\rho \frac{{ℰ}^{2}}{k}$$

where ℰ=ep

For turbulent viscosity:

(7) $${µ}_{\tau }=\rho C_{µ}\frac{k^{2}}{ℰ}$$

The coefficients for the turbulent viscosity, turbulent kinetic energy, and dissipation are fixed:$\;C_{ℰ1}=1.44,C_{ℰ2}=1.92,\delta_{k}=1,C_{µ}=0.09,\delta_{ℰ}=1.3$

The turbulent kinetic energy production can be expressed as:

(8) $$P_{K}={µ}_{\tau }\left[\nabla u:\left(\nabla u+{\left(\nabla u\right)}^{\tau }\right)-\frac{2}{3}{\left(\nabla \cdot u\right)}^{2}\right]-\frac{2}{3}\rho k\nabla \cdot u$$

where *ρ*: fluid density, *p*: pressure, *u*: fluid flow velocity, *I*: identity matrix, µ: dynamic viscosity of the fluid, τ: stress tensor, ∇: del operator, *k*: turbulent kinetic energy, ep: turbulent dissipation rate.

The velocity of the flowing fluid is nondimensionalized using the Reynolds number:

(9) Re=ρuDµ

The heat transfer characteristics are evaluated using the Nusselt number (Nu) through the local as well as global heat transfer:

(10) $${\mathrm{Nu}}_{\log}=\frac{k_{hnf}}{k_{bf}}\frac{\partial \theta }{\partial y}$$

(11) $${\mathrm{Nu}}_{\mathrm{avg}}=\frac{1}{L}\overset{L}{\underset{0}{\int }}Nu_{log}dL$$

Modeling of hybrid nanofluid:

In this study, the working fluid is taken as Al_2_O_3_-MWCNT hybrid-nanofluid, which is basically a suspension of Al_2_O_3_ and MWCNT nanoparticles in the base fluid (water) following the mixture rules [45]. The mixture of Al_2_O_3_ and MWCNT nanoparticles (known as hybrid nanoparticles) is chosen for the present thermal–hydrodynamic study. In general, the thermal conductivity as well as the electrical conductivity of Al_2_O_3_ are less compared to MWCNT. Al_2_O_3_, however, suffers from inferior conductivities compared to that of MWCNT, and due to the combined (hybrid) preparation of MWCNT and Al_2_O_3_ nanoparticles, superior conductivities (much higher than the base liquid) are obtained. In this connection, it is worth mentioning that the flow of hybrid nanofluids in the heat exchanger devices is a relatively newer area; therefore, more research works can give a greater understanding of it, which is required for real-life applications [45]. On the other hand, many recent works have considered MWCNT-Al_2_O_3_-water hybrid nanofluid, indicating a wide field of its applications and reliability. It motivates us to choose MWCNT-Al_2_O_3_-water hybrid nanofluid as a working fluid for this study. The hybrid nanoparticles’ volume fraction is expressed using the symbol ‘*φ*’. The properties of the hybrid nanofluid are estimated using the standard correlations, which are expressed as

$$\varphi =\varphi_{Al_{2}O_{3}}+\varphi_{MWCNT}$$

$$\rho_{np}=\frac{\varphi_{Al_{2}O_{3}}\rho_{Al_{2}O_{3}}+\varphi_{MWCNT}\rho_{MWCNT}}{\varphi }$$

(12) $${\left(C_{p}\right)}_{np}=\frac{\varphi_{Al_{2}O_{3}}{\left(C_{p}\right)}_{Al_{2}O_{3}}+\varphi_{MWCNT}{\left(C_{p}\right)}_{MWCNT}}{\varphi }$$

$$\beta_{np}=\frac{\varphi_{Al_{2}O_{3}}\beta_{Al_{2}O_{3}}+\varphi_{MWCNT}\beta_{MWCNT}}{\varphi }$$

$$k_{np}=\frac{\varphi_{Al_{2}O_{3}}k_{Al_{2}O_{3}}+\varphi_{MWCNT}k_{MWCNT}}{\varphi }$$

$$\sigma_{np}=\frac{\varphi_{Al_{2}O_{3}}\sigma_{Al_{2}O_{3}}+\varphi_{MWCNT}\sigma_{MWCNT}}{\varphi }$$

Hybrid Nanofluid:

$$\sigma_{hnf}=\left(1-\varphi \right)\sigma_{bf}+\varphi \sigma_{np}$$

$$\rho_{hnf}=\left(1-\varphi \right)\rho_{bf}+\varphi \rho_{np}$$

$${\left(\rho \beta \right)}_{hnf}=\left(1-\varphi \right){\left(\rho \beta \right)}_{bf}+\varphi {\left(\rho \beta \right)}_{np}$$

$${\left(\rho C_{p}\right)}_{hnf}=\left(1-\varphi \right){\left(\rho C_{p}\right)}_{bf}+\varphi {\left(\rho C_{p}\right)}_{np}$$

(13) $$\alpha_{hnf}=\frac{k_{hnf}}{{\left(\rho C_{p}\right)}_{hnf}}\;$$

$$\frac{k_{hnf}}{k_{bf}}=\frac{k_{np}+\left(n-1\right)k_{bf}-\left(n-1\right)\left(k_{bf}-k_{np}\right)\varphi }{k_{np}+\left(n-1\right)k_{bf}+\left(k_{bf}-k_{np}\right)\varphi }$$

$${µ}_{hnf}=\frac{{µ}_{bf}}{{\left(1-\varphi \right)}^{2.5}}$$

$$\frac{\sigma_{hnf}}{\sigma_{bf}}=1+\frac{3\left(\sigma_{np}-\sigma_{bf}\right)\varphi }{\left(\sigma_{np}+2\sigma_{bf}\right)-\left(\sigma_{np}-\sigma_{bf}\right)\varphi }$$

where the total entropy and efficiency are defined as:

(14) $$S_{tot}=\frac{k}{{\left(T_{avg}\right)}^{2}}\left[{\left(\frac{\partial T}{\partial x}\right)}^{2}+{\left(\frac{\partial T}{\partial y}\right)}^{2}\right]+\frac{{µ}_{nf}}{T_{avg}}\left[\frac{{ℰ}_{p}}{k}\left(u^{2}+v^{2}\right)+2{\left(\frac{\partial u}{\partial x}\right)}^{2}+2{\left(\frac{\partial v}{\partial y}\right)}^{2}+{\left(\frac{\partial u}{\partial y}+\frac{\partial v}{\partial x}\right)}^{2}\right]+\frac{\sigma_{nf}}{T_{avg}}\beta^{2}v$$

With

$$T_{avg}=\frac{T_{inlet}+T_{tube}}{2}$$

$$ℰp=\frac{q}{q_{max}}=\frac{q}{C_{min}\left(\theta_{inlet}-\theta_{tube}\right)}\;$$

$$q=UA\Delta T_{ml}\;$$

$$\Delta T_{ml}=\frac{\Delta T_{1}-\Delta T_{2}}{ln\left(\Delta T_{1}/\Delta T_{2}\right)}$$

$C_{min}$ = minimum heat capacity rate

*q* = heat transfer rate, kW

A = total heat transfer surface area of the heat exchanger and total heat transfer surface area of all matrices of a regenerator, m^2^

U = overall heat transfer coefficient, $W/m^{2}K$

Boundary conditions

The boundary conditions adopted for this study are:

1—symmetry (thermal insulation) at the region borders.

2—fixed inlet temperature T = 285 K with speed *v* = −0.01 m/s.

3—fixed temperature at the inner surfaces of the tube T = 278 K.

4—transport dominated by convection at the exit.

5—tube material: aluminum

The thermo-physical properties of the used hybrid nanofluids are presented in Table 1.

## 3. Numerical Methodology

The evolved Equations (1)–(7) of the chosen geometry are numerically solved utilizing the boundary conditions. The conservation equations are discretized over the computing domain following the Galerkin finite element method (FEM). The computing domain is split into an appropriate smaller mesh structure. The discretization of the computational domain is achieved through the function space adopting the piecewise cubic polynomials. The appropriate mesh elements are distributed in a manner such that all of the related hydro-thermal boundary layers nearer to the walls are captured correctly. The element size is selected through the mesh independence study. The elemental equations are computed through an iterative process (with convergence criteria of 10^−8^ for obtaining the final solution) and solved data are stored for post-processing, which are utilized for generating the local and global data.

### 3.1. Grid Test

**Figure 3:**
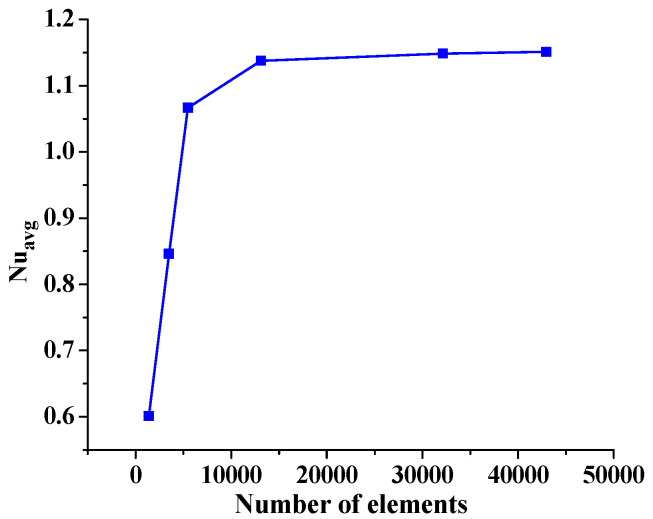
Grid test, variation of the average via element number.

Before conducting the extensive analysis of the present problem, an extensive grid independence study is carried out considering the circular tube bundle. Here, six different mesh sizes, extra coarse, very coarse, coarse, normal, fine, and very fine, are utilized with number elements of 1398, 3501, 5487, 13,108, 32,128 and 42,948. The comparison is made through the average Nusselt number, which is shown in Figure 3 and Table 2. From Figure 3 and Table 2, it is observed that the last three values of the average Nusselt are close to each other, so the middle value (with a fine mesh structure) is chosen to save the computational time to generate the correct results.

### 3.2. Validation

**Figure 4:**
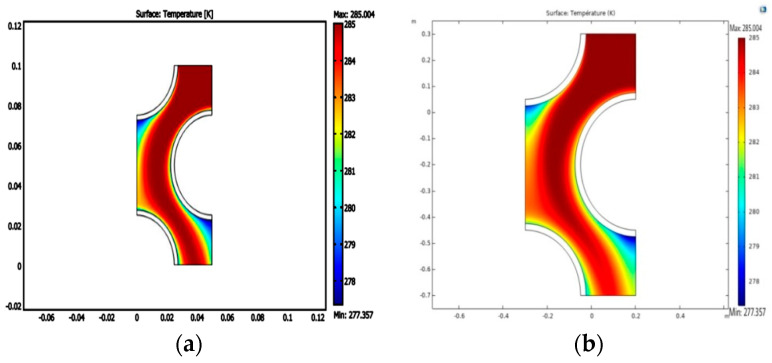
Validation of our results with ref. [47]. (**a**) Results of ref. [47]; (**b**) our results.

After finalizing the mesh structure, a validation study is conducted considering the published work of Roy and Mondal [47]. The comparison of the present findings and the published work of Roy and Mondal [47] is shown in Figure 4 using the isotherms. The comparison shows a close match between the two results [47].

## 4. Results and Discussion

The present study focuses on the improvements of the tube/shell heat exchanger to maximize the efficiency either by altering the geometrical shape of the tubes and/or by adding nanoparticles in its heat transfer fluid. Water-based Al_2_O_3_-MWCNT hybrid nanofluid is utilized here as a heat transfer fluid. The results are presented using the streamline, isotherms, and entropy contours, and the average Nusselt number for two main parameters: the Reynolds number (2400 ≤ Re ≤ 2700) and the volume fraction (0.01 ≤ *φ* ≤ 0.04), to study the heat transfer performance of the heat exchanger. The shape (circular, square, rectangular, and diamond) of the heat exchanger tube is modified from a conventional circular for obtaining the best shape.

### 4.1. Effect of Nanofluid Volume Fraction

**Figure 5:**
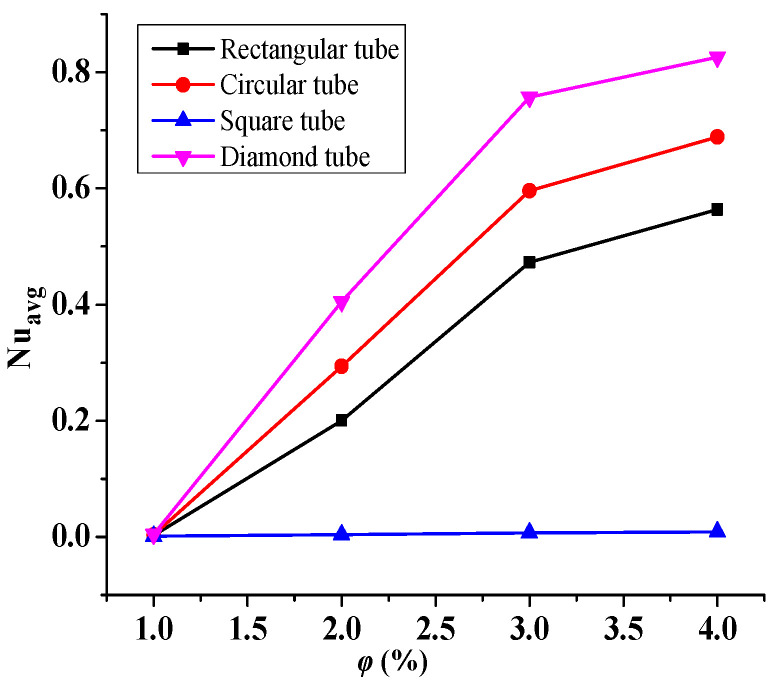
Variation of the Nu_avg_ with the increasing *φ*(℅) for Re = 2600.

The effect of the increasing hybrid nanofluid volume fraction (0.01 ≤ *φ* ≤ 0.04) on the heat transfer is shown in Figure 5, at Re = 2600 for the four different shapes of the tube bundle. From the figure, it is observed that the Nu_avg_ rises monotonically with the increasing *φ* for the circular, rectangular, and diamond-shaped tubes, whereas there is no change in the Nu_avg_ for the square-shaped tubes. With the growth in the *φ*, the effective thermal conductivity of the heat transfer fluid (compared to the base fluid) increases, which leads to the improvement in the heat transfer rate. It should be mentioned that these properties cause an enhancement in the thermal conducting properties of the working fluid [45,46,47,48,49,50,51,52,53,54]. Therefore, the average Nu is proportional to the nanoparticle concentrations, and such a correlation contributes to the enhanced convective transfer. Further improvement in the Nu_avg_ is accomplished due to the modification in the tube shape, which allows more removal of the heat from the tube bundles. However, a diamond-shaped tube only offers superior performance in heat transfer compared to all other shaped tubes.

### 4.2. Reynolds Number Effect

**Figure 6:**
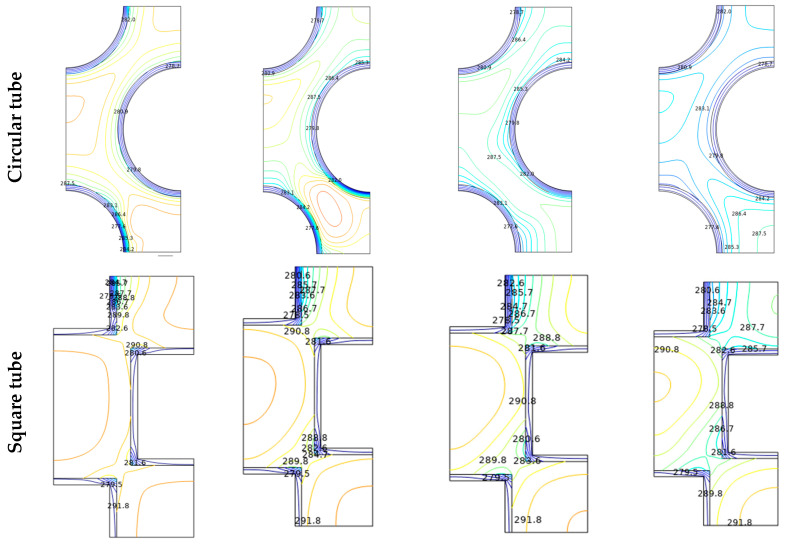

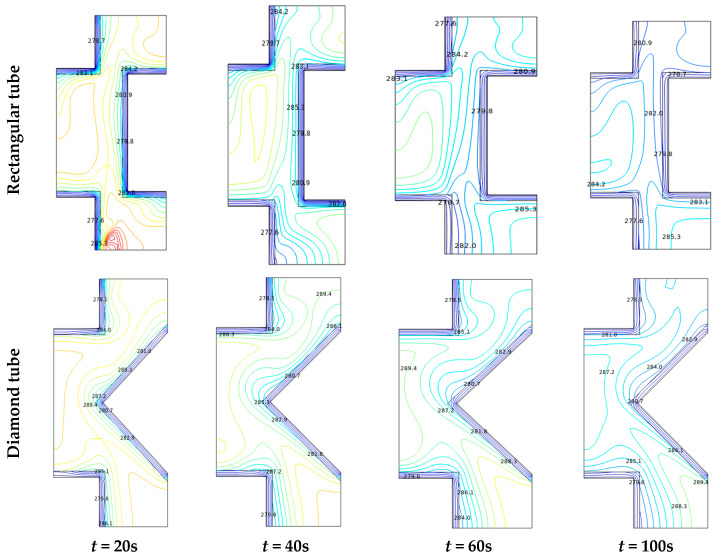
Isotherms in a tube/shell heat exchanger with different shape of manufacturing tubes for Re = 2600, *φ* = 0.02.

**Figure 7:**
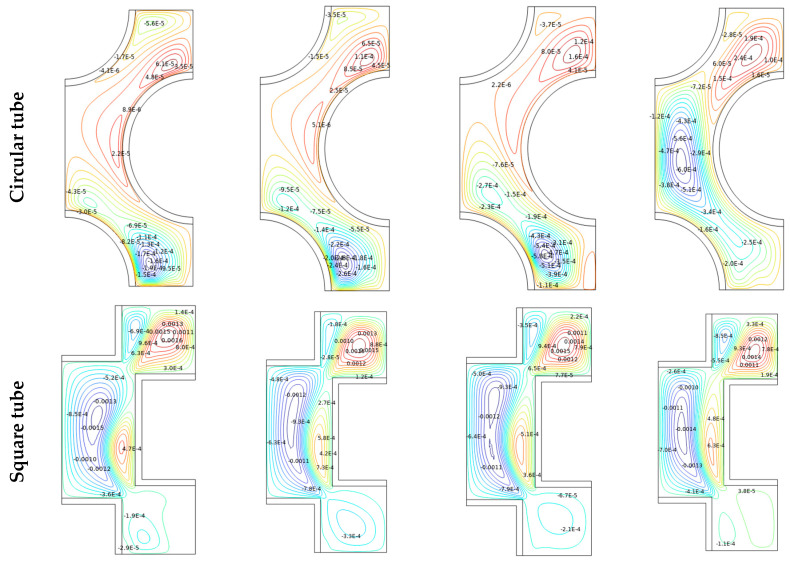

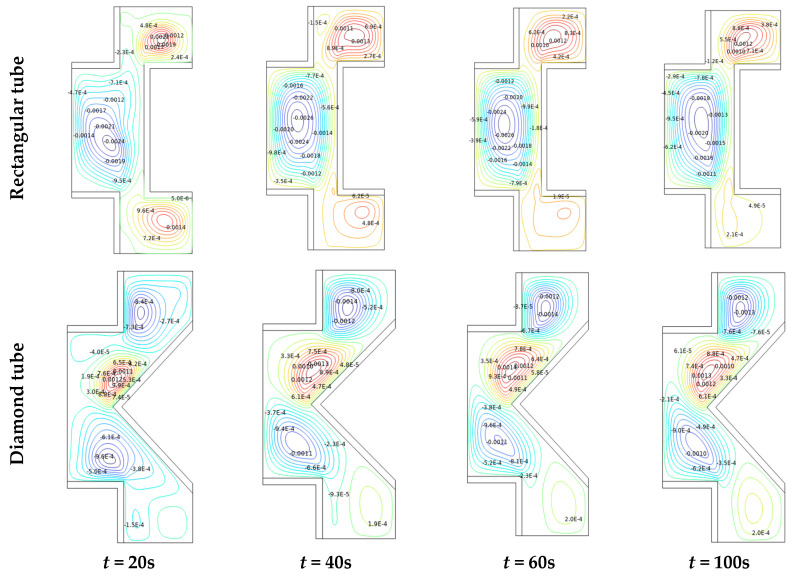
Streamlines in a tube/shell heat exchanger with different shape of manufacturing tubes for Re = 2600, *φ* = 0.02.

The reason for the improvement in the Nu_avg_ could be realized from the isotherm and streamline contours, as shown in Figure 6 and Figure 7, respectively, for the different time steps (*t* = 0 to 100 s) with Al_2_O_3_-MWCNT/water hybrid nanofluid at Re = 2600 and φ = 0.02. Here, the effects of the Reynolds number (2400 ≤ Re ≤ 2700) on the streamlines, isotherms, and entropy production are also analyzed. As the fluid flows through the complex path, the fluid flows through the narrow gaps due to the thermal buoyancy caused by the temperature gradient between the inlet wall and the outlet walls of the shell/tube heat exchanger. It is pertinent to mention that, at the initial time, the flows begin to move from the inlet (of the flow passage) and then move to the next complex and narrow passage in between the tube bundle gaps. The isotherms replicate the thermal conditions (temperature) across the chamber. As the fluid is heated from below, the concentration of the isotherm lines is clustered at the bottom and fades as it spreads vertically toward the outlet of the flow passage. Heat is absorbed by the working fluid from the bottom and dissipates as it rises to an outlet as it begins to spread upwards with the passage of time. At the lower flow regime (Re = 2600), the convection fluxes are very weak, and uniformly distributed temperature lines occupy the entire domain. Therefore, the thermal conduction modes dictate the thermal behavior. The values of the isothermal lines gradually increase from the hot wall to the cold wall. This topology is the thermal stratification, where thermal diffusion causes heat transfer. With the progress in time, the isotherm contours distribute uniformly over the flow passage, and such uniformity is more with the diamond-shaped tube bundle compared to all other shapes of the tubes. From the streamline contours (Figure 7), it is observed that there are multi-cellular vortices within the flow path. The position, size, and shape of these vortices change with progress in time. In fact, the results reveal that over time there is a linear growth in the flow functions, which is due to the flow through the complex narrow gaps. Figure 7 shows that over time, the eddies in the vicinity of the entrance wall become larger and stronger, and it soon heads upwards, allowing strong free convection, which strengthens the flow velocity and accelerates the fluid flow. With the increasing Re, as the flow passes through the inlet for increased Re values, the isotherms were blown to the edge. Furthermore, in the case of rectangular and square tubes, the fluid faces more resistance in its flow path due to the presence of the shape corners at the inlet and outlet of the flow domain. However, the circular and diamond fluid faces lower resistance, which leads to a higher rate of heat removal.

**Figure 8:**
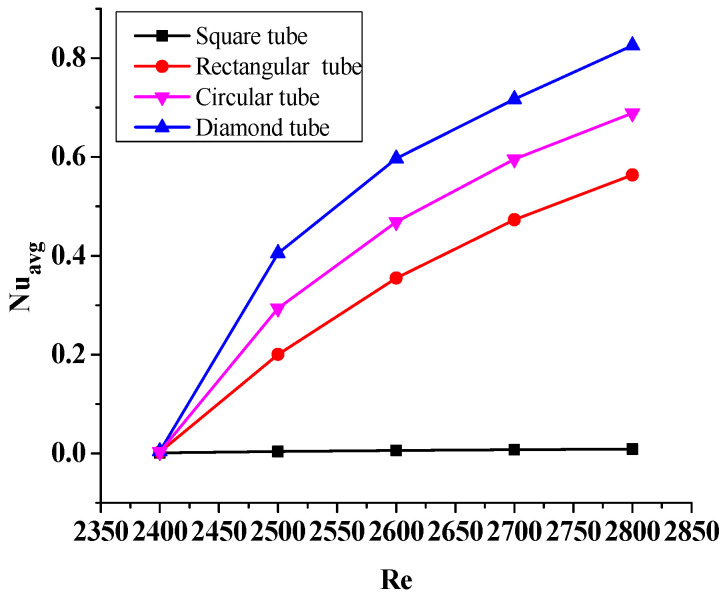
Variation of the Nu_avg_ as a function of change in the Re at φ = 0.02.

For understanding the overall impact of the different shaped tube bundles on the overall heat transfer, the Nu_avg_ is plotted for the varying Re, which is shown in Figure 8. The heat transmission efficiency of Al_2_O_3_-MWCNT/water hybrid nanofluid improves as the flow velocity increases, which enhances the thermal convection effect. The higher fluid Re corresponds to the higher heat transfer. Furthermore, the diamond-shaped tube bundles correspond to the superior heat transfer, and this is true for any Re values.

### 4.3. The Effect of Tube Shape

By examining the results of streamlines and isotherms as shown in Figure 6 and Figure 7, the flow velocity is lessened with the square-, circular- and rectangular-shaped tubes due to the flow separation compared to the diamond-shaped tubes, which increases the transfer of heat since it provides the largest flux function values. In fact, the vortex is distributed between the inlet wall and the outlet wall, providing a higher area for the flowing fluid, thus enhancing the rate of heat transfer. The variation in the temperature also goes a long way in improving buoyancy forces, which stimulate natural convection.

Additionally, Figure 5 and Figure 8 show that the pipe’s diamond shape improves the heat transfer by exhibiting the maximum peak of average Nu values in comparison to the other shapes. Furthermore, the geometric characteristics of the diamond shape and the uniform area available around it, which allow hybrid nanofluid dispersion. This helps to improve the Nu_avg_ number and thus thermal convection.

### 4.4. Entropy Generation

**Figure 9:**
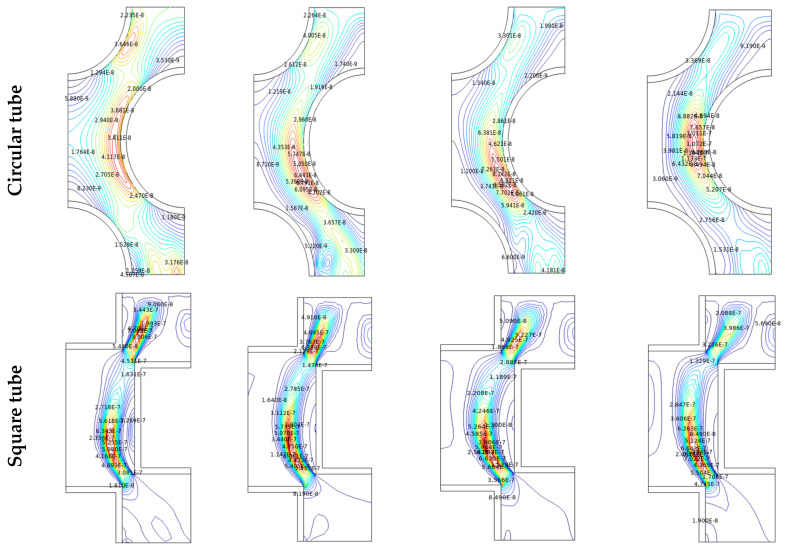

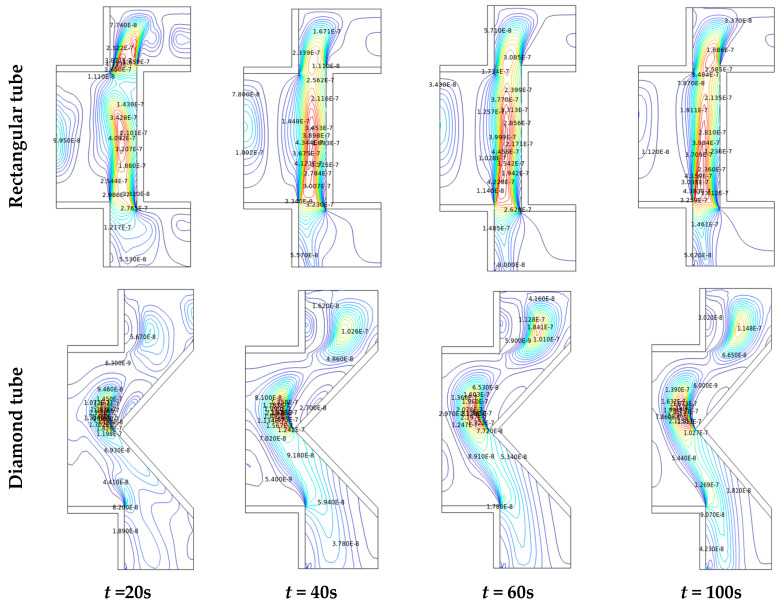
Entropy in a tube/Shell heat exchanger with different shape of manufacturing tubes for Re = 2600 and φ = 0.02.

**Figure 10:**
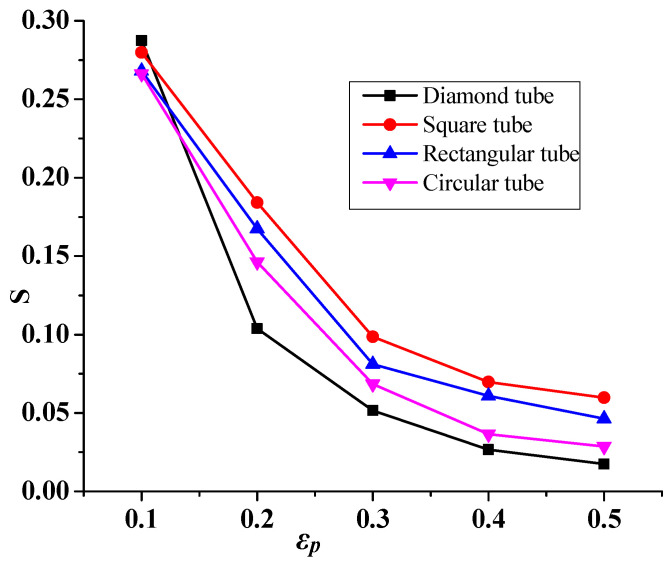
Entropy generation (S) variation as a function of effectiveness of the heat exchanger (*ε_p_*) for Re = 2600 and φ = 0.02.

In this section, entropy generation is calculated (Figure 9), and low entropy is observed at the hot wall cones, as a result of weaker isotherms close to the region. Therefore, more circulation of fluid is essential. The distribution is also bi-cellular, except the shape of the circulating cells, and it follows an oval shape. This implies an enhanced induced flow. Therefore, thermal convection dominates, which is indicated by the isotherms. The latter indicates enhanced convection, which favors the mechanism of convection over that of conduction. Moreover, Figure 10 shows that the diamond tube shape improves the heat exchanger’s effectiveness by exhibiting the highest decreases in entropy compared to the other shapes.

## 5. Conclusions

The present study focuses on the improvements of the tube/shell heat exchanger to maximize the efficiency either by altering the geometrical shape of the tubes and/or by adding nanoparticles in its heat transfer fluid. Water-based Al_2_O_3_-MWCNT hybrid nanofluid is utilized here as a heat transfer fluid. The analysis is carried out for two main parameters: the Reynolds number and the volume fraction, to study the heat transfer performance of the heat exchanger. The shape (circular, square, rectangular, and diamond) of the heat exchanger tube is modified from a conventional circular for obtaining the best shape. The following conclusions were revealed:

- The heat transfer rises with the growing fluid velocity (function of Re).
- The heat transfer rises with growing nanoparticle concentration. Compared to the circular tubes, the heat transfer enhancement goes up to 103.07% with a particle concentration of 2%.
- The diamond-shaped tube only offers superior performance in heat transfer compared to all other shaped tubes.
- The diamond tube shape improves the heat exchanger performance by exhibiting the most decreases in entropy compared to the other shapes.

In general, the outcome of the study is very significant in the industrial field and can solve many heat transfer problems. However, the present study could be extended for further research to incorporate three-dimensional models, other geometries, and flow-controlling parametric ranges and boundary conditions for unsteady or turbulent fluid flow, which are limited in the present study.

## Figures and Tables

**Table 1:** Thermo-physical proprieties of the Al_2_O_3_-MWCNT/water [44,46].

	*ρ* $\left(\mathrm{kg}/m^{3}\right)$	*k* (W/m k)	$C_{p}\;(J/\mathrm{kg}\;k)$	*σ* (s/m)
Water	997.1	0.613	4179	5.5 × 10^−6^
MWCNT	2100	3000	711	10^−7^
** $Al_{2}O_{3}$ **	3950	36.96	785.02	

**Table 2:** Mesh comparison.

Mesh	Element Number	Average Nusselt Number
Extra coarse	1398	0.6011
Very coarse	3501	0.84613
Coarse	5487	1.0666
normal	13,108	1.13758
Fine	32,128	1.14863
Very fine	42,948	1.15084

## Data Availability

Data are available on request.

## Abbreviations

**Nomenclature**	
g	Acceleration due to gravitational (m·s^−2^)
H	Heat exchanger height (m)
$c_{p}$	Specific heat capacity at constant pressure (J/(kg·K))
Nu	Nusselt number (average)
*p*	pressure (Pa)
Pr	Prandtl number
Re	Reynolds number
*t*	time (s)
T	temperature (K)
q	heat flux by conduction (W/m^2^)
$q_{r}$	heat flux by radiation (W/m^2^)
*u*, *v*	components of velocity (m s^−1^)
x, y	Cartesian coordinates (m)
**Greek symbols**	
α	thermal diffusivity (m^2^·s^−1^)
*β*	coefficient of thermal expansion (K^−1^)
μ	dynamic viscosity (N m^−2^·s^−1^)
ν	kinematic viscosity (m^2^·s^−1^)
ρ	fluid density (kg m^−3^)
φ	hybrid nanoparticles volume fraction (%)
ψ	Dimensionless stream function
*θ*	Dimensionless temperature
τ	viscous stress tensor (Pa)
∇	del operator
